# Physical and psychological aspects of multiple sclerosis: Revisiting the Multiple Sclerosis Impact Scale (MSIS-29)

**DOI:** 10.1177/13524585241288393

**Published:** 2024-10-30

**Authors:** Carolyn A Young, David J Rog, Basil Sharrack, Radu Tanasescu, Seema Kalra, Suresh K Chhetri, Lisa Wilde, Roger J Mills, Alan Tennant

**Affiliations:** Institute of Systems, Molecular and Integrative Biology, University of Liverpool, Liverpool, UK; The Walton Centre NHS Foundation Trust, Liverpool, UK; Manchester Centre for Clinical Neurosciences, Northern Care Alliance NHS Foundation Trust, Salford, UK; Department of Neurology, University of Sheffield, Sheffield, UK; Nottingham Centre for Multiple Sclerosis and Neuroinflammation, Department of Neurology, Nottingham University Hospitals NHS Trust, Nottingham, UK; Academic Unit of Mental Health and Clinical Neuroscience, University of Nottingham, Nottingham, UK; University Hospitals of North Midlands NHS Trust, Stoke-on-Trent, UK; Lancashire Teaching Hospitals NHS Foundation Trust, Preston, UK; North West Anglia NHS Foundation Trust, Peterborough, UK; Institute of Systems, Molecular and Integrative Biology, University of Liverpool, Liverpool, UK; The Walton Centre NHS Foundation Trust, Liverpool, UK; Leeds Institute of Rheumatic and Musculoskeletal Medicine, University of Leeds, Leeds, UK

**Keywords:** Rasch, multiple sclerosis, MS Impact Scale-29, trajectory model, minimal important change, minimal detectable change, TONiC study

## Abstract

**Background:**

The MSIS-29 measures the physical and psychological impact of MS.

**Objective:**

The associations between MSIS-29 domains and demographic/clinical aspects were examined and trajectories analysed over time.

**Methods:**

Data were collected in the Trajectories of Outcome in Neurological Conditions study for a diverse population of people with MS, with follow-up for up to 5 years. Following Rasch analysis, minimal important change (MIC) was computed for ensuing total, physical and psychological domains.

**Results:**

Fit to the Rasch model using data from 5921 participants validated physical, psychological and total domains, and the conversion table transforms raw scores to interval-level metric equivalents. These domains showed significant differences across demographic (age, gender, employment, education, and marital status) and clinical (subtype, treatment, and duration) factors with large effect sizes. The MIC scores were physical: 9.1, total: 14.1, which were both above measurement error, and psychological: 5.5 which was not, so 1.6% of participants reported psychological change which was clinically important but not statistically significant. Trajectory analysis showed three groups, one stable and two with significant slopes, improving and deteriorating.

**Conclusion:**

The MSIS-29 has shown adequate fit to the Rasch model after accommodating problems with local item dependency, through a bi-factor solution. The domains showed good discrimination across key factors.

## Introduction

Multiple sclerosis (MS) is a complex disease whose many symptoms impact upon disability, mood and quality of life.^1^ Almost 42% of participants report reduced ability to perform daily activities, as well as negative effect on emotional and social factors.^2^

It follows that physical and psychological functioning are two important traits to be considered in people with MS (pwMS). The MSIS-29 is a self-administered measure with 20 items covering physical aspects and 9 items covering psychological aspects,^3^ which is reported to have high test–retest reliability and internal consistency.

This study examines the MSIS-29 in a large cohort of pwMS, looking at construct validity using the Rasch model, and other aspects such as minimal important change (MIC). It examines the association between the domains and key demographic and clinical aspects, the converted interval-level metrics are then used to explore the trajectory of domains over time.

## Methods

### Main sample

Participants were recruited into the Trajectories of Outcome in Neurological Conditions-MS (TONiC-MS) study https://www.finders-study.org/tonic where eligibility criteria included adults with MS (by revised McDonald criteria^4^) of any subtype and disability level.

Disease subtypes at study entry were classified as relapsing remitting (RR), primary progressive (PP) or secondary progressive (SP). Duration since diagnosis and Expanded Disability Status Scale (EDSS) band were recorded from medical records.^5^ Disease-modifying therapies (DMT) were categorised as low or high efficacy.^6^ Written informed consent was obtained from all participants prior to enrolment. Ethical approval was granted from research committees (reference 11/NW/0743).

### Longitudinal sample

Further questionnaire packs were sent at approximately 9-month intervals. At each follow-up, as well as repeating the questionnaire pack, respondents were asked to comment whether their disability and worry levels were worse, the same, or better compared to when they last completed a pack.

### Calibration sample

Construct validity was examined using the Rasch measurement model.^7^ To facilitate the analysis, a sample of 1000 was drawn from the full sample’s first three time points, and further randomised into two sub-samples of 500 for training and validation analyses. No individual was included more than once in the sample.^8^ The sample size of each sub-sample was consistent with retaining a Type 1 error rate of 5% using the RUMM2030 software.^9,10^

### Outcome measures

Several patient-reported outcome measures (PROMs) were included in the pack in addition to the change scores on disability and worry. The questionnaires relevant to the current investigation are:

1. MS Impact Scale (MSIS-29) – The 29 items in MSIS-29 (v1) measure impact in five levels (not at all, a little, moderately, quite a bit, extremely) where respondents are asked to record ‘the impact of MS on your day-to-day life during the past 2 weeks’. Total score ranges 0–116, physical score ranges 0–80, and psychological score ranges 0–36. Higher scores indicate greater impact.
2. Hospital Anxiety and Depression Scale (HADS) – Two subscales measuring anxiety and depression have associated clinical cut points delivering none-possible-probable caseness.^11^

### Statistical analysis plan

An overview of the application of the Rasch model is given in Tennant and Kucukdeveci,^12^ details in Supplemental File 1 for this analysis. Differential item functioning (DIF) refers to items that function differently between groups of participants: although the participants have the same level of the factor being measured, they answer the scale item differently.^13^

One way analysis of variance is applied to examine the discrimination across EDSS (for physical) and HADS caseness (for psychological). Should a total score be derived, this will be tested against EDSS.

The standard error of measurement (SEM) and the smallest detectable difference (SDD) of the MSIS-29 domains are calculated from baseline data. The minimal detectable change (MDC) is the minimum change in score for an individual that must occur to be sure that the change is not just due to measurement error.^14^ The MIC reflects the smallest change in score that pwMS perceive as meaningful.^15^ The MDC and MIC are determined from longitudinal data. The MIC used an anchor-based method, based on the patients’ perceived change of disability for the physical domain and of worry for the psychological domain.^16^ It was calculated as the largest of the upper (when positive) or lowest (when negative) 95% confidence interval for the mean differences between before and after scores in the two groups rated as either ‘worse’ or ‘better’ by the respondents.^17,18^ For the change variables, the MDC and MIC can be combined to produce a four-fold classification.^19^ This will identify groups where change was (1) not statistically significant or important (<MDC < MIC); (2) significant but not important (>MDC < MIC); (3) important but not significant (>MIC < MDC); and (4) both significant and important (>MDC > MIC). Effect sizes of the various estimates are reported. All values are calculated on the interval metric.

Using the metric transformation of each domain, a group-based trajectory model (GBTM) is applied in the full data set to ascertain if there were groups displaying different trajectories over time.^20^ Details of GBTM methods are in Supplemental File 1.

## Results

### Sample descriptions

#### Cross-sectional data

Mean age at baseline in the full sample of 5921 pwMS was 50.2 years (SD 12.0), mean duration of MS was 11.1 years (SD 9.8), 73.8% were female. 66% were RR subtype, 22.9% were SP, and 11.2% PP. Over half (51.3%) were EDSS 4 or below (independently ambulant); 37% were EDSS 4.5–6.5, 11.4% were EDSS 7–9.5, and 0.25% unknown. There was a significant difference in EDSS level by disease subtype with, for example, EDSS 0–4 ranging from 8.8% in SP, to 71.4% in RR (Chi-square χ^2^ 2.1e + 03(9); *p* < 0.001). Information on DMT use was available for 5633 (95.1%) of participants. Overall, 44.2% were on DMT, including 59.7% of those with RR, 15.3% of SP and 3.6% of PP. The most widely used DMT was an interferon (see Table 1). Within RR, 39.2% were on low efficacy DMT, 20.5% high efficacy.^6^

**Table 1.** Tabulation of disease-modifying therapies at consent.

DMT at Consent	DMT efficacy			
	None or unclassified	Low	High	% of total
None	3145	0	0	55.83
Alemtuzumab	0	0	102	1.81
Azathioprine	2	0	0	0.04
Cladribine	0	0	6	0.11
Dimethyl fumarate	0	409	0	7.26
Fingolimod	0	0	226	4.01
Glatiramer	0	472	0	8.38
Interferons	0	644	0	11.43
Mitoxantrone	3	0	0	0.05
Natalizumab	0	0	477	8.47
Ocrelizumab	0	0	6	0.11
Siponimod	3	0	0	0.05
Stem cell transplant	5	0	0	0.09
Teriflunomide	0	56	0	0.99
Laquinimod in clinical trial	5	0	0	0.09
Other agents in clinical trial	55	0	0	0.98
Daclizumab (now withdrawn)	0	0	17	0.30
Total	3218	1581	834	100

DMT: disease-modifying therapies.

#### Longitudinal data

Data from 2416 pwMS who had at least completed their baseline and first follow-up questionnaires were analysed. Mean time from baseline to first follow-up was 22.6 months (SD 13.2), median time 19.6 months (interquartile range [IQR]: 10.7–31.4). Mean age was 50.7 years (SD: 11.5); MS duration 11.0 years (SD: 9.8); 65.0% RR, 23.1% SP and 11.9% were PP. There were no significant differences in MS subtype between those followed-up and the remainder of the full sample (χ^2^ 3.07, df(2); *p* = 0.215). Around 75.1% were female, and 45.6% were on DMT.

#### Calibration sample

The calibration sample displayed no significant difference to those remaining in the full sample across age group, gender, MS subtype, duration group, DMT, or EDSS levels (χ^2^ > 0.05).

#### Fit to the Rasch model

**Figure 1.**
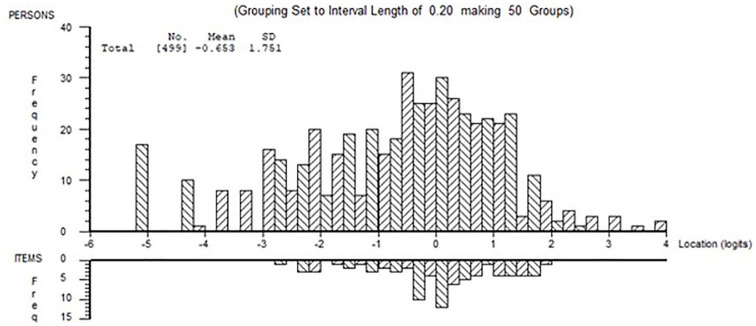
Person-item threshold distribution of physical domain in training sample. Showing distribution of person estimates (above the x-axis) and item threshold estimates (below the x-axis).

Fit of physical and psychological domains to the Rasch model were examined in the calibration sample. Full details are given in Supplemental File 2. Briefly, the person-item (threshold) distribution of the 20 physical items in the training sample is shown in Figure 1. The scale is reasonably well targeted, although weak at the lower end of physical disability, with a floor effect shown between −4.2 and −5.2 logits. Item transition from ‘Not at all’ to ‘A little’ (threshold 1) is mostly observed at the lower impact level, while the transition from ‘Quite a bit’ to ‘Extremely’ (threshold 4) is at the high impact end. The item in which movement away from ‘Not at all bothered by’ is most easily achieved was ‘Do physically demanding tasks’. In contrast, the item ‘Difficulty moving about indoors’ was the least likely to transfer from ‘Quite a bit’ to ‘Extremely bothered’. There is no particular item order across this range of measurement.

There were several disordered thresholds associated with the transition from ‘A little’ to ‘Moderately’. There were breaches of local item independence which had to be accommodated, for example ‘I worry about how I will cope with the future’ and ‘Despite my difficulties I still manage to cope with daily life’ had a residual correlation of 0.483. Following this, fit to the model was achieved using a testlet approach, with the first 10 items grouped as ‘physical’ and the remaining 10 items grouped as ‘participation’ (e.g. ‘Limitations in your social and leisure activities at home’). The result was replicated in the validation sample.

In the psychological domain, fit to the model was poor. While the person-item distribution was adequate, three pairs of items were locally dependent. With an average residual correlation of −0.11, the pair of items ‘Feeling mentally fatigued’ and ‘Problems concentrating’ displayed a residual correlation of 0.182. ‘Feeling mentally fatigued’ was also the easiest item regarding moving away from ‘Not at all bothered’. The item ‘Feeling depressed’ was the one where the transition from ‘Quite a bit’ to ‘Extremely’ was the most difficult to achieve. Disordered thresholds were present in six out of nine items with the transition between ‘A little’ to ‘Moderately’ the source of the problem. Clustering local dependent items into ‘super items’ (i.e. post hoc following LD analysis) achieved fit.

Examining whether a total score from all 29 items was viable, fit was poor in the training sample. Principal component analysis of the residuals split the item set by domain, resulting in 33.6% of *t*-tests < 5%. Inspection of the item set and the pattern of local dependency suggested there were item clusters which were conceptually linked (e.g. items 1–4 physical; items 25–29 mood). Grouping these sets into two testlets, each combining sets of physical and psychological items, resulted in good fit to the model where just 3% of the variance needed to be discarded (Supplemental file 2, Table S1). Of note, the distribution of items and persons for the total score was more inclusive of the range of impact.

In the validation sample, the results were replicated other than DIF appeared for subtype, age, and duration. As those with SP differed in that they tend to be older and with longer duration, subtype was split for SP and the person estimates derived from the unsplit and split solutions compared. The p value of the paired *t*-test of the difference was 0.1043, so the unsplit solution was retained. The DIF for age and duration was no longer evident after subtype was split.

Conversion of raw scores to interval metric for all three domains is given in Table 2.

**Table 2.** Conversion table to convert raw scores to interval-level metric for MSIS-29 total, physical and psychological domains.

Raw score	Total	Physical	Psychological
0	0.0	0.0	0.0
1	8.3	7.4	3.4
2	13.6	12.1	5.8
3	17.0	15.2	7.5
4	19.6	17.5	8.8
5	21.6	19.4	10.0
6	23.5	21.0	11.1
7	25.1	22.4	12.1
8	26.6	23.8	13.0
9	28.1	25.0	13.9
10	29.5	26.1	14.7
11	30.8	27.2	15.4
12	32.1	28.2	16.1
13	33.3	29.1	16.8
14	34.5	30.0	17.4
15	35.7	30.9	18.0
16	36.8	31.7	18.6
17	38.0	32.5	19.1
18	39.0	33.2	19.6
19	40.1	33.9	20.2
20	41.1	34.6	20.7
21	42.1	35.2	21.2
22	43.1	35.8	21.7
23	44.0	36.3	22.2
24	44.9	36.8	22.7
25	45.8	37.3	23.3
26	46.7	37.8	23.9
27	47.5	38.2	24.5
28	48.3	38.7	25.1
29	49.1	39.1	25.8
30	49.9	39.4	26.5
31	50.6	39.8	27.3
32	51.3	40.1	28.2
33	52.0	40.5	29.3
34	52.7	40.8	30.8
35	53.3	41.0	32.9
36	54.0	41.3	36.0
37	54.6	41.6	
38	55.2	41.9	
39	55.8	42.1	
40	56.3	42.4	
41	56.9	42.6	
42	57.4	42.9	
43	57.9	43.2	
44	58.4	43.4	
45	58.9	43.7	
46	59.4	43.9	
47	59.9	44.2	
48	60.3	44.5	
49	60.8	44.8	
50	61.2	45.1	
51	61.6	45.4	
52	62.0	45.7	
53	62.4	46.1	
54	62.8	46.4	
55	63.2	46.8	
56	63.6	47.2	
57	64.0	47.6	
58	64.4	48.1	
59	64.8	48.6	
60	65.1	49.1	
61	65.5	49.6	
62	65.9	50.1	
63	66.2	50.7	
64	66.6	51.4	
65	67.0	52.0	
66	67.3	52.7	
67	67.7	53.5	
68	68.1	54.2	
69	68.4	55.1	
70	68.8	55.9	
71	69.2	56.9	
72	69.6	57.9	
73	70.0	59.0	
74	70.4	60.3	
75	70.8	61.7	
76	71.2	63.3	
77	71.6	65.4	
78	72.0	68.3	
79	72.4	72.8	
80	72.9	80.0	
81	73.3		
82	73.8		
83	74.3		
84	74.7		
85	75.2		
86	75.7		
87	76.3		
88	76.8		
89	77.4		
90	77.9		
91	78.5		
92	79.1		
93	79.7		
94	80.4		
95	81.0		
96	81.7		
97	82.4		
98	83.1		
99	83.8		
100	84.6		
101	85.4		
102	86.2		
103	87.0		
104	87.9		
105	88.8		
106	89.7		
107	90.8		
108	91.9		
109	93.1		
110	94.4		
111	96.0		
112	97.8		
113	100.1		
114	103.3		
115	108.2		
116	116.0		

Instructions for use of the conversion table for MSIS-29 v1.

Providing the respondent has answered all the items, take the raw score and look across to the interval scale estimate for the relevant domain.

For example, if you are converting the total score, a raw score of 100 would give a standardised metric total score of 84.6.

A raw physical score of 40 gives a standardised metric of 42.4.

A raw psychological score of 35 gives a standardised metric of 32.9.

#### Descriptives, discrimination and detection

The parameter estimates for the three domains, physical, psychological, and total, were exported into the main data set for analysis. Metric domain levels for demographic and clinical characteristics are shown in Table 3. Most domains displayed significant differences across demographic and clinical factors. However, with such a large sample, statistical significance was often generated where the actual difference was small, for example, effect size of the significant difference between those married/cohabiting, or not, on physical domain was 0.10 and psychological domain 0.16, both considered trivial. In contrast, the total score across the age gradient has an effect size of 0.62, considered medium. Difference in physical function between high and low DMT has an effect size of just 0.22, considered (very) small. Difference of level of physical functioning of those on high-efficacy DMT, and those not on any DMT, was 0.24.

**Table 3.** Descriptive statistics of the metric MSIS-29 in the baseline sample: physical, psychological and total domains. *N* = 5795.

Attribute	Physical		Psychological		Total		N
	Mean	SD	Mean	SD	Mean	SD	
Age (years)							
<42	31.9	15.7	15.1	7.7	47.5	21.0	1342
42–49	37.5	14.3	15.6	7.2	53.5	19.5	1376
50–57	40.7	13.4	15.8	7.0	56.9	18.4	1470
58+	43.0	11.5	15.1	6.9	59.1	16.3	1584
Gender							
Male	39.6	14.1	15.1	17.2	55.3	18.9	1513
Female	38.1	14.4	15.4	7.2	54.2	19.3	4259
Onset							
PP	40.1	10.8	15.8	6.7	61.9	15.3	632
RR	34.3	14.3	14.8	7.3	49.5	19.6	3818
SP	47.7	9.5	16.7	6.9	65.4	13.6	1295
Duration (years)							
<3	33.9	14.8	15.1	7.3	49.4	19.8	1180
3–8	36.3	14.9	15.2	7.3	52.1	20.1	1609
9–14	40.1	13.8	15.6	7.2	56.3	18.9	1355
14+	43.1	11.9	15.6	6.9	59.5	16.5	1524
DMT							
No	40.7	13.8	15.6	7.1	57.0	18.6	3196
Yes	35.8	14.4	15.1	7.3	51.4	19.6	2576
Employment							
No	44.0	11.6	16.6	7.0	61.2	16.2	3226
Part	34.3	13.8	14.6	6.9	49.3	18.6	1102
Full	30.0	14.8	13.3	7.2	43.9	19.7	1512
Education							
<HND	40.4	13.4	16.0	7.0	56.9	18.2	2318
HND+	37.3	14.8	14.9	7.2	52.9	19.8	3522
Marital status							
Married	38.3	14.5	15.0	7.2	53.9	19.5	3920
Other	39.7	13.6	16.2	7.1	56.5	18.4	1405
Total	38.5	14.3	15.3	7.2	54.5	19.2	5795

SD: standard deviation; PP: primary progressive; RR: relapsing remitting; SP: secondary progressive; DMT: disease-modifying therapy; HND: higher national diploma.

The discriminant validity (effect size) of the three domains is shown for relevant comparator measures in Table 4, strong significant gradients were found for every domain. MDC and MIC of the various domains are also shown.

**Table 4.** Discriminant ability (effect size) of the MSIS-29 at baseline together with MDC and MIC from first follow-up.

	Physical	Psychological	Total
EDSS			
0–4	30.6		44.6
4.5–6.5	45.5		63.5
7.0–7.5	51.2		69.9
8+	52.6		70.0
ANOVA p	<0.001		<0.001
Effect Size EDSS low -> high	1.84		1.62
HADS-Anxiety			
No		11.4	47.4
Possible		17.4	58.1
Probable		21.7	65.7
ANOVA p		<0.001	<0.001
Effect Size No -> probable		1.78	1.13
HADS-Depression			
No		12.7	47.8
Possible		19.2	64.8
Probable		23.1	73.0
ANOVA p		<0.001	<0.001
Effect Size No -> Probable		1.76	1.63
Cross-Sectional			
SEM	3.8	2.7	4.7
SDD	10.5	7.4	13.4
%OR	13.1	20.7	11.4
Longitudinal change			
SEM^c^	1.7	2.0	2.8
MDC	4.8	5.7	7.6
MDC as %OR	6.0	15.8	6.6
MIC	9.1	5.5	14.1
Confidence Interval*	2.6 to −6.5	2.2 to −3.3	3.8 to −10.3
MIC as %OR	11.4	15.3	12.2

EDSS: Expanded Disability Status Scale; HADS: Hospital Anxiety and Depression Scale; SEM: standard error of measurement; SDD: smallest detectable difference; %OR: percent of operational range of scale; SEM^c^: standard error of measurement of change score; MDC: minimal detectable change; MIC: minimal important change.

* Confidence intervals of the worse and better estimates used to generate the MIC.

In the longitudinal data, 41.2% reported their disability had worsened, whereas 53.3% reported that it had stayed the same, 5.5% reported improvement. Worry was the same for 66.9%, worse for 24.7% and improved for 8.5%. Using the metric transformation of the various domains, Table 5 shows the distribution of the MDC and MIC. Both the physical and total scales can fully identify the MIC, but for the psychological scale, there were 1.6% respondents where the change was important but could not be distinguished from measurement error.

**Table 5.** Pattern of important and significant changes in MSIS-29 based upon the Minimal Detectable Change and Minimal Important Change.

MSIS-29-Physical	Frequency	Percentage (%)	Cumulative
Not important or significant	1303	54.75	54.75
Not important but significant	634	26.64	81.39
Important and significant	443	18.61	100.00
Total	2380	100.00	
MSIS-29-Psychological			
Not important or significant	1786	75.20	75.20
Important and significant	552	23.24	98.44
Important but not significant	37	1.56	100.00
Total	2375	100.00	
MSIS-29-Total			
Not important or significant	1360	57.46	57.46
Not important but significant	603	25.48	82.93
Important and significant	404	17.07	100.00
Total	2367	100.00	

#### Trajectory analysis

**Figure 2.**
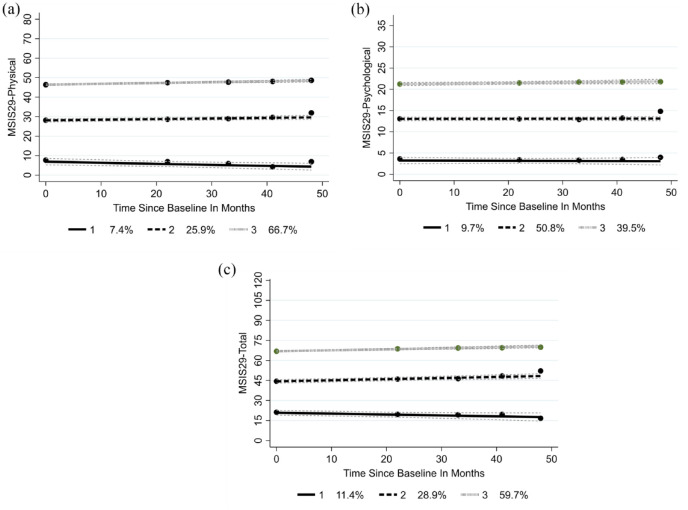
(a) MSIS-29 physical trajectories, (b) MSIS-29 psychological trajectories and (c) MSIS-29 total trajectories.

Three groups were identified meeting the criteria specified in Supplemental File 1. Following physical and psychological aspects over 5 years, both physical and total domains showed small numbers (group 1: 7.4% for physical trajectories and 11.4% for total trajectories) with a low level of functioning which slightly improved (Figure 2(a) and (c)). In the physical domain, group 3 (66.7%) had a significant worsening over time. In the total domain, group 2 (28.9%) showed a significant worsening while 59.7% showed no significant increase over the follow-up. There was no significant movement in the three groups identified in the psychological domain (Figure 2(b)).

## Discussion

This study supports the construct validity of the MSIS-29 through fit to the Rasch measurement model, having accommodated local item dependencies. The physical and psychological domains were confirmed, though there was a limitation in the physical domain at the lower end of the scale, as observed previously.^21^ A total domain was also identified, which showed no limitation across the full impact experienced by pwMS.

The domains showed strong discrimination across the comparator measures and most clinical and demographic factors, although effect sizes were often trivial. All three domains showed the ability to identify the MIC, albeit with a small proportion of the psychological domain being undifferentiated from measurement error. The MIC for the physical domain is 9.1 when appropriately calculated on the metric. Earlier work using receiver operating characteristic curves for EDSS range 5.5–8 found an MIC of 8.^22^ In the trajectory analysis, both the physical and total domains showed a small group improving over time and a much larger group who worsened. The psychological groups remained stable over the 5 years follow-up.

The MSIS-29 demonstrated some problems, notably the disordered thresholds in many of the items, also identified in previous studies applying the Rasch model to MSIS-29 data.^23,24^ However, there is inconsistency in these reports with a community sample reporting disordered thresholds and lack of evidence for a total score,^25^ while a clinical trial sample reported fully ordered thresholds.^21^ A further clinical trial reported ordered thresholds and suggested that the scale could be restructured into three domains, effectively splitting the physical domain into ‘symptoms’ and ‘general limitations’ item sets.^24^ This study split the physical item set into ‘physical’ and ‘participation’ groups, based on the conceptual basis of the International Classification of Functioning, Disability and Health (ICF).^26^ The ‘physical’ items are a mix of impairments, or physical symptoms, and activity limitations. The difference in this study is that by applying the bi-factor structure, these two item sets worked as a single domain with little loss of variance. Previous work suggested that the range of impact covered by physical domain items did not match the patient range of impact, particularly for patients with lower impact.^21,24^ This study supported that finding for the physical scale but showed no such shortfall for the measurement range for the total scale.

Our findings and earlier studies suggest that item thresholds from RR clinical trials work as intended, but not in community studies with varied subtypes. This raises the question as to whether the domains are invariant by MS subtype. In this study, while there was some lack of invariance, mostly driven by the SP subtype, these did not prove to be substantial. However, these analyses were run on testlets where some invariance may have been accommodated.^27^

This study shows that the MSIS-29 is suitable for both clinical and epidemiological use providing the raw scores are transformed to the interval-level metric using Table 2. This is important as the local dependency and associated multidimensionality of the MSIS-29 has been resolved by the bi-factor solutions underlying Table 2. The physical and total scores show little floor or ceiling effect, and their MIC are well above the MDC or ‘noise’ of the scale at 9.1 (MDC 4.8) and 14.1 (MDC 7.6), respectively. Thus for these domains, all changes considered important by pwMS should be detectable. The physical domain requires just 6% and the total score 6.6% of operational range before error is overcome. Furthermore, the trajectory analysis indicates that the physical and total scores can track groups of pwMS who are stable, worsen or improve. These characteristics demonstrate the value of the interval metrics for epidemiological research and clinical care. Clinicians and researchers require to be able to detect clinically relevant change and distinguish between real change and measurement error. Knowing what level of change is significant for patients is critical for interpreting the impact of an intervention producing change.

In contrast, the psychological domain had an MIC at 5.5 which was less than the MDC (5.7) and thus meaningful change to the pwMS can occur within measurement error; our data showed a small number of pwMS reporting important change which would be undetected as falling within the MDC. The MDC was 15.8% of the operational range so many changes might have to be disregarded as falling within measurement error. In research, a much larger sample would be required to show change on the MSIS-29 psychological scale. The trajectory analysis using the psychological domain could detect distinct groups of pwMS who entered the study with very different levels of psychological impact from their MS, half showing some impact and about 40% more severely impacted. About 10% had little psychological impact. These three groups remained at stable levels of impact.

Future work should address whether the MIC is stable across different subtypes of MS, particularly for the psychological domain.^28^ Any factors which predispose pwMS to fall into the improving group for MSIS-29 merit investigation as they may suggest improvements to clinical care. The stability of trajectories of the psychological domain requires more investigation.

In conclusion, the total, physical and psychological scores for the MSIS-29 can easily be converted to interval-level measurement, thus permitting parametric analyses such as change scores and trajectory examination. Clinicians and researchers may be confident in the measurement precision and discriminant ability of the MSIS-29, both in its subscales and total score. Clinically important change (MIC) for the physical and total scores is low and above measurement error (MDC), but the psychological score is less robust. The impact of MS varies within a large population of pwMS with different subgroups showing physical and total domain trajectories which remain stable, worsen or sometimes improve.

## Supplemental Material

sj-pdf-1-msj-10.1177_13524585241288393 – Supplemental material for Physical and psychological aspects of multiple sclerosis: Revisiting the Multiple Sclerosis Impact Scale (MSIS-29) Supplemental material, sj-pdf-1-msj-10.1177_13524585241288393 for Physical and psychological aspects of multiple sclerosis: Revisiting the Multiple Sclerosis Impact Scale (MSIS-29) by Carolyn A Young, David J Rog, Basil Sharrack, Radu Tanasescu, Seema Kalra, Suresh K Chhetri, Lisa Wilde, Roger J Mills and Alan Tennant in Multiple Sclerosis Journal
