# Supplementary material for: Physical and psychological aspects of multiple sclerosis: Revisiting the Multiple Sclerosis Impact Scale (MSIS-29)
Source: Mult Scler. 2024 Oct 30;30(13):1630–41. doi: 10.1177/13524585241288393 (PMC11568641; doi:10.1177/13524585241288393)
Supplement: sj-pdf-1-msj-10.1177_13524585241288393 – Supplemental material for Physical and psychological aspects of multiple sclerosis: Revisiting the Multiple Sclerosis Impact Scale (MSIS-29) [file sj-pdf-1-msj-10.1177_13524585241288393.pdf]

Supplemental file for

Physical and psychological aspects of multiple sclerosis: revisiting the Multiple Sclerosis Impact Scale (MSIS-29)

Carolyn A Young, David J Rog, Basil Sharrack, Radu Tanasescu, Seema Kalra, Suresh K Chhetri, Lisa Wilde, Roger J Mills, Alan Tennant, on behalf of the Trajectories of Outcome in Neurological Conditions-MS Study Group
Multiple Sclerosis Journal 2024

Correspondence to: CA Young. Email: Cayoung@liverpool.ac.uk
ORCID: https://orcid.org/0000-0003-1745-7720

Contents

Supplementary File 1 1
Why Rasch Measurement Theory 1
Methods of Rasch analysis of the MSIS-29 3
Group-based trajectory model 5
Supplementary File 2 6
Detailed Results of fit to the Rasch Model 6
Table 1. Fit of MSIS-29 to the Rasch model 9
References 10

In this supplementary file we explain why it is necessary to apply Rasch Measurement Theory to the MSIS-29, before detailing the methods of the Rasch analysis and the group-based trajectory model. Next we provide detailed results of Rasch analysis.

Supplementary File 1

Why Rasch Measurement Theory

While the original publication for the MSIS-29 used factor analysis, a later publication found only partial support for the two-factor hypothesised model[1]. An earlier paper had investigated the scale from a Rasch Measurement Theory (RMT) perspective and supported

a two-factor structure but not a total score[2, 3]. Another study applied both classical and modern test theory in relapsing remitting (RR) MS and found that both subscales demonstrated robust measurement properties using classical, Item Response Theory (IRT), and Rasch techniques[4]. Since that time the RMT framework has increasingly offered a much more comprehensive integrated approach to the evaluation of Patient Reported Outcome Measures (PROM). It is worth noting that the recommendations for future research in the definitive publication by Hobart and colleagues stated that "as traditional psychometric methods were used to develop and evaluate the MSIS-29, it is also important that newer psychometric methods such as Rasch and Item Response Theory models are used to evaluate the MSIS-29." [5].

Factor analysis is essentially a method for examining the structure of an instrument to determine dimensionality, that is, construct validity[6]. While some aspects of PROM evaluation can be investigated by factor analysis, there are some, particularly for polytomous scales such as the MSIS-29, which can only be seen through RMT. Specifically, monotonicity of item responses, that is identifying the response order of the categories of polytomous items that are not associated with an increase in the underlying trait, referred to as disordered thresholds. This analysis provides insights into how the scale is working at the item level, and if certain category options are causing problems, sometime leading to a revision of the scale responses.

The measurement of reliability is also integral to the RMT approach, which can be monitored over any adjustments that are necessary. Such adjustments are often necessary due to a breach of the local item (in)dependence requirement (LID), a breach of which is where two items are correlated after conditioning on the total score. RMT provides an instant overview of LID whereas factor analysis would require identifying correlation of individual errors, not always acceptable to some in a Confirmatory Factor Analysis (CFA). RMT provides a residual correlation matrix to identify such breaches and allows for the development of testlets or super items, where items are grouped to overcome the problem. These are just aggregations of sets of items which are identified as locally dependent. If there is an a priori grouping such as a subscale, it is preferred to call these testlets, whereas super items are ad hoc groupings identified by the residual correlation matrix, and may consist of just two items added

together. A key aspect of this and the integrated approach is that LID inflates reliability, and thus the decrease in reliability is annotated at each step necessary to overcome any problem with LID[7].

Another important aspect of RMT is that it allows for an evaluation of the targeting of the instrument. A recent publication found a shortfall for the MSIS-29 in evaluating changes associated with Disease Modifying Therapies in patients with minimal disability[8].

Overall, there is the desire to achieve fundamental measurement and the requirements which follow. This means that the measurement of person ability is independent of the distribution of items in the scale, and also the calibration of item difficulty is independent of the distribution of the ability of persons in the sample. Out of all the models within the general IRT framework, the Rasch model is unique because it is the only parametric model where the raw score over all items is a sufficient statistic for the person parameter. Due to this property, conditional maximum likelihood (CML) estimation can be used to estimate item parameters consistently without assuming a specific population distribution for the latent trait. Furthermore, at the core of the Rasch model is the notion of homogeneity, that same ordering of items and persons irrespective of their level on the trait. So, for persons, regardless of where they are on the trait being measured, the ordering of items will always be the same, a requirement for fundamental measurement. This is not the case for other parametric IRT models, nor for factor analysis.

Finally, applying RMT can contribute to the latest developments in test equating, where scales measuring the same attribute are placed upon the same reference metric. This allows comparison across studies which use different scales for the same attribute, so facilitating meta-analysis[9].

Methods of Rasch analysis of the MSIS-29

Data were tested upon a calibration sample of 1000 cases divided into training and validation samples. As local item independence (LD) has been shown to be influential in its effect upon fit, dimensionality and threshold ordering, this was initially tested where residual correlations with a value of 0.2 above the average values (which may be negative) were deemed a breach

of that requirement[10-12]**.** Where LD was detected, items were grouped into 'super items' (i.e. post hoc)[13] or testlets (i.e. a priori)[14].

Item (super item or testlet) fit was tested by a (conditional) Chi-square where fit was deemed acceptable at >0.05 (Bonferroni adjusted). Where items are grouped in some fashion, the proportion of variance retained to reach a solution is reported as the Expected Common Variance[15]. Where this is reported a value less than 0.7 is indicative of requiring a multidimensional model, a value above 0.9 a unidimensional model, and the grey area in between, undetermined, requiring further evidence[16]. The correlation between resulting clusters is also reported. Should the analysis result in just two clusters of items, then a conditional Ch-Square test of fit becomes available in the RUMM2030 software[17].

Unidimensionality was tested according to Smith, testing two item sets based upon the principal component analysis of the residuals[18]. Less than 5% of these differences must be found to support unidimensionality. Domains were also tested for invariance (Differential Item Functioning, DIF) by a series of contextual factors including age (grouped), gender, time (repeated measurement), duration of MS since diagnosis (grouped) and onset type. If DIF was observed, relevant items were split across the contextual factor, and the impact of DIF tested by the effect size of the difference of split- and unsplit- person estimates. If the effect size was above 0.1, DIF was deemed to be substantial[19].

Reliability was reported as both a 'Person Separation Index (PSI) and Cronbach's Alpha. They are both based upon the same formulae but Cronbach's alpha is calculated on the ordinal while PSI on the metric. If the data are normally distributed then the PSI and α will be similar, but otherwise PSI will deviate (usually lower) if the data are skewed. This is because the PSI is much more a measure of the ability to discriminate units of measurement, and the more skewed and clustered the scores (e.g. towards the floor of the scale), the less measurement units can be differentiated.

Parameters from the calibration sample were then imported into the main data set to obtain estimates for each domain. Where acceptable fit was achieved, a transformation was made of the raw total score to an interval scale score equivalent to the range of the ordinal score.

That is, the logit value of each raw score point from zero to the maximum permitted for each scale is transformed to the same operational range of the scale, that is, zero to the maximum, but with a single decimal point to indicate it is a transformed metric.

Group-based trajectory model

The time metric was the median month since the baseline questionnaire at each follow-up. Each domain was assessed at baseline and up to four further follow-ups, and modelled with a censored normal distribution. The number and shape (via polynomial functions) of trajectories were determined by analysing one to five group models without covariates. To accommodate attrition, a 'dropout' model was applied, specified in its basic form of constant dropout across assessment occasions[20]. The Bayesian Information Criterion (BIC) was used to determine the best-fitting model, also with consideration for a useful and parsimonious model. Average posterior probabilities above 0.7 were also deemed to indicate optimal fit[21]. Missing data were handled using a maximum likelihood approach based on a missing-at-random assumption.

Supplementary File 2

Detailed Results of fit to the Rasch Model

Fit of the physical and psychological domains to the Rasch model were then examined in the calibration sample. The scale is well targeted, covers five logits, and the item transition from ’Not at all’ to ’A little’ (threshold 1) is mostly observed at the lower impact level of the scale, while the transition from ‘Quite a bit’ to ‘Extremely’ (threshold 4) can be found at the high impact end of the scale. There is no particular item order across this range of measurement and there is very little floor and ceiling effect. Examination of the threshold ordering found several disordered thresholds associated with the transition from ’A little’” to ’Moderately’.

Initial fit of the physical subscale in the training sample was poor (Table 1:Training-Physical-Basic). Substantial breaches of the local independent assumption were evident, with thirteen pairs of dependent items, clustering within the first ten and second ten items. For example, the items “I worry about how I will cope with the future” and “Despite my difficulties I still manage to cope with daily life” had a residual correlation of 0.483. Furthermore, there was a scattering of DIF across all contextual factors other than the use of disease modifying therapies. For example, the item “Carry things” showed DIF by age and MS subtype. No action was taken at this stage of the analysis for either threshold disordering or DIF, due to the extensive breach of the local item independence assumption.

Initially, from the pairs of clustered items, six groups of dependent items could be observed. These were grouped into six super items, leaving just five unique items. Fit to the model remained poor (Table1:Training-Physical-Super item). DIF was still evident, and four items had the same pattern of disordered thresholds. On closer inspection of the item content, the first ten items, which were all clustered together in 4 super items, were all physical, whereas the second ten (including 2 super items), were predominately participation. Consequently, two concept-based testlets were created which showed excellent fit to the Rasch model, including a conditional chi-square test of fit (Table1:Training-Physical-Testlet). The Explained Common Variance (ECV) indicated that just three percent of the variance was discarded under the bi-factor solution, and that the two testlets had a correlation of 0.94, consistent with a parallel

form. There was no DIF by any contextual factors other than MS subtype. Here, those with RR appeared to have less impact on the participation testlet. Comparison of the unsplit and split item estimates did show a significant difference (paired t-test p=0.029), but the effect size of the difference was 0.03, and so no further action was taken. The thresholds of the two testlets were fully ordered which is to be expected as the expected value curve of the testlet will always be monotonic with the total score because it is a property of the model irrespective of thresholds.

This bi-factor solution was tested in the validation sample. This was fully replicated, along with DIF by MS subtype (Table 1:Validation-Physical-Testlet). In this instance it was not possible to differentiate subtypes from the Item (Testlet) Characteristic Curve, and so no further action was taken. 97% of the variance was retained in this solution, with the latent correlation between the two testlets at 0.94 (Table 1:Validation-Psychological).

In the psychological domain, fit to the model was again poor (Table 1:Training-Psychological-Basic). Three pairs of items were locally dependent. With an average residual correlation of -0.11, the pair of items “Feeling mentally fatigued” and “Problems concentrating” displayed a residual correlation of 0.182. Disordered thresholds were present in six out of nine items with the transition between ’A little’ to ‘Moderately’ the source of the problem.

Grouping the locally dependent items into two super items, with the remaining items as unique, gave an improved fit to the model (Table 1:Training-Psychological-Super Item). DIF was present for SP and those aged 58 years and over on the item “Feeling irritable, impatient, or short-tempered”. Both characteristic curves followed the same pattern, with the response to these items lower when psychological distress was higher. The item was split for MS subtype, which showed a significant difference between the unsplit and split solutions, but had an effect size of that difference of 0.06, and so the unsplit solution was retained. Only 7% of the variance was discarded to obtain an interval latent estimate for this scale under this solution. However, fit was marginal, and so conceptually based testlets were created (worry, anxiety, depression vs rest), where fit improved substantially (Table 1:Training-Psychological-Testlet). Fit improved, and there was no DIF and unidimensionality, However on this occasion the solution required 14% of the variance to be discarded. As such the super-item solution

was retained. This solution was fully replicated in the validation sample, albeit once again with weak fit (Table 1:Validation-Psychological-Testlet).

Consideration was then given as to whether a total score from all 29 items was viable. In the training sample, fit was poor (Table 1:Training-Total- Basic). Principal component analysis of the residuals split the item set by domain, resulting in 33.6% of t-tests < 5%. A closer inspection of the item set, and the pattern of local dependency, suggested that there were clusters of items which were largely conceptually based which may be tested (e.g. items 1-4 physical; items 25-29 mood). Grouping these sets into two testlets, each combining sets of physical and psychological items, resulted in good fit to the model where just 3% of the variance needed to be discarded (Table 1:Training-Total-Testlet).

In the validation sample, the results was replicated other than DIF appeared for subtype, age and duration (Table 1:Validation-Testlet). Those with SP differed. As SP tend to be older and with longer duration, subtype was split for SP and the person estimates derived from the unsplit and split solutions compared. The p value of the paired t-test of the difference was 0.1043, and so the unsplit solution was retained. The DIF for age and duration was no longer evident after subtype was split.

Table S1. Fit of MSIS-29 to the Rasch model

Sample/	Residual SD		Chi-Square Fit			Reliability		Differential Item	Dimensionality	ECV	Correl
Domain	Item	Person	Value	df	P	PSI	α	Functioning	t-test% > 0.05		-ation
**Training Sample (n=500)**											
Physical											
Basic	3.421	1.416	416.6	180	<0.001	0.95	0.97	Subtype/EDSS/Age/ Gender	11.9	-	-
Super item	3.566	1.152	192.0	90	<0.001	0.92	0.93	Subtype/EDSS/Age/ Gender	5.8	0.95	0.80
Testlet	0.011	0.725	62.9	72	0.789	0.90	0.94	Subtype	2.2	0.97	0.94
Psychological											
Basic	2.921	1.368	156.9	81	<0.001	0.87	0.92	Subtype/Age	2.8	-	-
Super item	2.439	1.209	75.0	54	0.031	0.84	0.85	Subtype/Age	1.0	0.93	0.82
Testlet	3.382	0.759	29.3	28	0.398	0.83	0.79	-	1.4	0.86	0.93
Total Score											
Basic	3.523	1.634	702.3	261	<0.001	0.95	0.97	Age/Gender/Subtype/ Duration/EDSS		-	-
Testlet (Conceptual)	1.791	0.963	104.8	98	0.298	0.94	0.94	-	4.4	0.97	0.97
**Validation Sample (n=500)**											
Physical /Testlet	0.148	0.790	65.4	72	0.696	0.93	0.93	Subtype	1.5	0.96	0.92
Psychological /Super item	2.230	1.188	82.5	54	0.007	0.84	0.86	Age	0.5	0.93	0.84
Psychological /Testlet	3.545	0.840	36.1	27	0.114	0.79	0.79	-	2.6	0.86	0.93
Total Score /Testlet	1.907	0.936	82.0	97	0.863	0.94	0.94	Subtype/Age/Duration	3.1	0.97	0.99

SD: standard deviation; df: degrees of freedom; PSI: person separation index; [illegible]: Cronbach's [illegible]; ECV: expected common variance; EDSS: Expanded Disability Status Scale

References

1. Fitzgerald SM, Li J, Rumrill PD, et al. Examining the factor structure of the Multiple Sclerosis Impact Scale. *Work (Reading, Mass)* 2014; 49: 523-538. 2013/07/05. DOI: 10.3233/wor-131669.
2. Rasch G. *Probabilistic Models for Some Intelligence and Attainment Tests*. Chicago: The University of Chicago Press, 1980.
3. Ramp M, Khan F, Misajon RA, et al. Rasch analysis of the Multiple Sclerosis Impact Scale (MSIS-29). *Health and Quality of Life Outcomes* 2009; 7: 58. DOI: 10.1186/1477-7525-7-58.
4. Bacci E, Wyrwich K, Phillips G, et al. Analysis of the psychometric properties of the Multiple Sclerosis Impact Scale-29 (MSIS-29) in relapsingremitting multiple sclerosis using classical and modern test theory. *Multiple Sclerosis Journal Experimental, Translational and Clinical* 2016; 2: 1-13. DOI: https://doi.org/10.1177/2055217316673235.
5. Hobart J, Lamping D, Fitzpatrick R, et al. The Multiple Sclerosis Impact Scale (MSIS-29): a new patient-based outcome measure. *Brain* 2001; 124: 962-973. 2001/05/04. DOI: https://doi.org/10.1093/brain/124.5.962.
6. Tavakol M and Wetzel A. Factor Analysis: a means for theory and instrument development in support of construct validity. *International journal of medical education* 2020; 11: 245-247. 2020/11/11. DOI: 10.5116/ijme.5f96.0f4a.
7. Oltmanns JR and Widiger TA. Maladaptive Variants of Adaptive Traits and Bloated Specific Factors. *J Res Pers* 2018; 76: 177-185. 2019/03/25. DOI: 10.1016/j.jrp.2018.08.006.
8. Regnault A, Loubert A, Brennan R, et al. Does the Multiple Sclerosis Impact Scale-29 (MSIS-29) have the range to capture the experience of fully ambulatory multiple sclerosis patients? Learnings from the ASCLEPIOS studies. *Mult Scler J Exp Transl Clin* 2023; 9: 20552173231201422. 2023/10/02. DOI: 10.1177/20552173231201422.
9. Prodinger B, Coenen M, Hammond A, et al. Scale Banking for Patient-Reported Outcome Measures That Measure Functioning in Rheumatoid Arthritis: A Daily Activities Metric. *Arthritis Care Res (Hoboken)* 2022; 74: 579-587. 2020/11/06. DOI: 10.1002/acr.24503.
10. Christensen KB, Makransky G and Horton M. Critical values for Yen's Q3: Identification of local dependence in the Rasch model using residual correlations. *Applied psychological measurement* 2017; 41: 178-194. 2018/06/09. DOI: 10.1177/0146621616677520.
11. Lundgren Nilsson Å and Tennant A. Past and present issues in Rasch analysis: the functional independence measure (FIM™) revisited. *J Rehabil Med* 2011; 43: 884-891.
12. Fennessy LM. *The impact of local dependencies on various IRT outcomes*. University of Massachusetts Amherst, 1995.
13. Baghaei P. A comparison of three polychotomous Rasch models for super-item analysis. *Psychological Test and Assessment Modeling* 2010; 52: 313.
14. Wainer H and Kiely G. Item clusters and computer adaptive testing: A case for testlets. *J Educ Meas* 1987; 24: 185-202.
15. Andrich D. Components of variance of scales with a bifactor subscale structure from two calculations of alpha. *Education Measurement: Issue and Practice* 2016; 35: 25-30.
16. Quinn H. *Bifactor Models, Explained Common Variance (ECV), and the Usefulness of Scores from Unidimensional Item Response Theory Analyses*. Masters Thesis, University of North Carolina at Chapel Hill, North Carolina, 2014.
17. Andrich D, Sheridan B and Luo G. RUMM2030: an MS Windows computer program for the analysis of data according to Rasch unidimensional models for measurement Perth, Western Australia: RUMM Laboratory, 2015.
18. Smith E. Detecting and evaluating the impact of multidimensionality using item fit statistics and principal component analysis of residuals. *J Appl Meas* 2002; 3: 205-231.
19. Rouquette A, Hardouin JB, Vanhaesebrouck A, et al. Differential Item Functioning (DIF) in composite health measurement scale: Recommendations for characterizing DIF with meaningful

consequences within the Rasch model framework. *PLoS One* 2019; 14: e0215073. 2019/04/10. DOI: 10.1371/journal.pone.0215073.
20. Haviland AM, Jones BL and Nagin DS. Group-based trajectory modeling extended to account for nonrandom participant attrition. *Sociological Methods & Research* 2011; 40: 367-390. DOI: 10.1177/0049124111400041.
21. Nagin DS and Odgers CL. Group-based trajectory modeling in clinical research. *Annual review of clinical psychology* 2010; 6: 109-138. 2010/03/03. DOI: 10.1146/annurev.clinpsy.121208.131413.
